# Pre-Digested Protein Enteral Nutritional Supplementation Enhances Recovery of CD4^+^ T Cells and Repair of Intestinal Barrier in HIV-Infected Immunological Non-Responders

**DOI:** 10.3389/fimmu.2021.757935

**Published:** 2021-12-24

**Authors:** Shi-Tao Geng, Jian-Bo Zhang, Yue-Xin Wang, Yu Xu, Danfeng Lu, Zunyue Zhang, Ju Gao, Kun-Hua Wang, Yi-Qun Kuang

**Affiliations:** ^1^ National Health Commission (NHC) Key Laboratory of Drug Addiction Medicine, First Affiliated Hospital of Kunming Medical University, Kunming Medical University, Kunming, China; ^2^ Scientific Research Laboratory Center, First Affiliated Hospital of Kunming Medical University, Kunming, China; ^3^ Department of Gastrointestinal and Hernia Surgery, First Affiliated Hospital of Kunming Medical University, Kunming, China; ^4^ Department of Dermatology, Second People’s Hospital of Dali City, Dali, China; ^5^ School of Medicine, Yunnan University, Kunming, China

**Keywords:** HIV/AIDS, immunological non-response, enteral nutrition, intestinal barrier function, inflammation

## Abstract

AIDS patients with immune non-response are prone to malnutrition, intestinal barrier damage, thus aggravating chronic immune activation and inflammation. However, nutritional interventions targeting malnutrition may be beneficial to restore immune function, improve clinical outcomes, and reduce mortality remains largely unclear. This work aimed to evaluate the efficacy of a nutritional supplement in HIV-infected immune non-responders (INRs). The subjects received oral supplementation of a pre-digested protein nutrition formula for three months. We show that the CD4^+^ T and CD8^+^ T cell counts were significantly increased after supplementation of the pre-digested enteral nutritional supplement. Among all pro-inflammatory cytokines in the serum, only IL-1β level was significantly decreased, while TNF-β was significantly increased (*P* < 0.05). The levels of intestinal mucosal damage markers, diamine oxidase (DAO), D-lactic acid (D-lactate), and lipopolysaccharide (LPS), decreased significantly (*P* < 0.05) after the nutritional intervention. Moreover, at month 3 after the intervention, the body weight, body mass index, albumin, and hemoglobin of all subjects were significantly increased (*P* < 0.05). The correlation analysis demonstrated a significantly negative correlation of CD4^+^ T cell count with levels of DAO (r = -0.343, *P* = 0.004), D-lactate (r = -0.250, *P* = 0.037), respectively, and a significantly positive correlation of IL-1β level with levels of DAO (r = 0.445, *P* < 0.001), D-lactate (r = 0.523, *P* < 0.001), and LPS (r = 0.622, *P* < 0.001). We conclude that the pre-digested enteral nutrition supplement is effective for HIV-infected INRs.

## Introduction

Antiviral therapy (ART) could significantly inhibit the human immunodeficiency virus (HIV). However, even if under ART and sustained virological suppression, a certain portion of HIV-infected individuals still cannot achieve complete immune reconstitution (1–3), they are called immune non-responders. The immune non-responders are prone to opportunistic infections and usually have a poor prognosis. Apart from acquired immunodeficiency syndrome (AIDS) -related diseases and deaths, the probability of non-AIDS-related diseases and deaths are higher for those with immune non-response (4–6).

Recent theoretical developments have revealed that the potential intermediate pathway for excessive early mortality in HIV-infected immunological non-responders due to malnutrition (7). Thus, nutrition plays an important role in the immune reconstitution among patients with HIV/AIDS. Besides direct damage of HIV to the immune system, HIV-positive individuals are prone to malnutrition due to inadequate dietary intake, nutritional losses, metabolic changes, and increased demand for macronutrients and micronutrients (8, 9). The integrity of the intestinal barrier is essential for human absorption of nutrients and health. Persistent intestinal mucosal barrier damage affects nutrient absorption (10), thereby leading to malnutrition. In addition, malnutrition also affects the integrity of the intestinal barrier and the balance of intestinal microbes (11, 12), further hindering the reconstruction of immune function.

Considering the important role of nutrition in immunity, a large number of studies have made efforts to improve the nutritional status of AIDS patients. Studies have shown that enteral nutritional supplementation is beneficial for the improvement of nutritional status and the recovery of CD4^+^ T cells (13–18). In addition, the composition of nutrition supplements, patient characteristics, and treatment methods vary greatly in different intervention studies. However, there is still a lack of relevant research on nutritional intervention for patients with immunological non-response.

Here, we proposed a pre-digested enteral nutritional supplement and evaluated its clinical effects on AIDS patients with immunological non-response by detecting serum levels of immune indicators and pro-inflammatory cytokines. Meanwhile, we also evaluated the effects of the pre-digested nutritional supplement on the intestinal barrier function and nutritional status in these patients.

## Methods

### Patients

The subjects of the study were AIDS patients with poor immune reconstitution recruited in the Second People’s Hospital of Dali City. The inclusion criteria were as follows: (1) Diagnosed as HIV/AIDS patients; (2) Male or female, aged ≥ 18 years old; (3) Plan to use nutritional support therapy for ≥ 3 months; (4) HIV infected persons or AIDS patients with nutritional therapy indications; (5) Patients with immune non-response: sustained HIV viral load < 50 copies/ml and CD4^+^ T cell count ≤ 350 cells/μL under ART over two years (19); and (6) Informed consent of patients/relatives to participate in this study. Exclusion criteria were as follows: (1) Contraindications to enteral nutrition in patients; (2) Patients with Apache II score > 10; (3) There are serious liver, kidney, heart, brain and other dysfunction, or there are serious complications such as hypertension, diabetes, coronary heart disease, *etc.*; (4) Participating in other clinical studies; (5) Known to be during pregnancy or lactation period; and (6) Those who have poor compliance and cannot cooperate with nutritional intervention. A total of 66 patients were receiving ART. A three-month nutritional intervention was conducted from July 2019 to November 2019.

This study was approved by the review board of the First Affiliated Hospital of Kunming Medical University (No. 2018-L-43), and written informed consent was obtained from the study participants. All experiments were performed in accordance with the approved guidelines and regulations according to the principles expressed in the Declaration of Helsinki, and the experimental protocols were approved by the institutional review board of the Kunming Medical University. The Chinese Clinical Trial Registry number of this work is ChiCTR2000037839 (http://www.chictr.org.cn/showproj.aspx?proj=35035).

### Study Design

The Enteral nutrition formula is given for three months, once a day for a nutrition supplement of 200 mL/200 kcal/day, which is supplemented in the form of an additional meal (the best before bedtime). Fasting blood samples were collected after one to three months of nutritional intervention. The serum was immediately separated by centrifugation and kept at -80°C until analysis. At the same time, body composition analysis and laboratory examinations were carried out during follow-up.

### Nutritional Assessment

Nutritional status was determined by body composition assessment and biological parameters. Body composition measurements were performed by using a multiple-frequency bioelectrical impedance model InBody S10 (Biospace, Seoul, Korea). The Inbody S10 was originally used for the measurements of medical purposes. As one of the latest impedance analyzers, it can be used for evaluating the nutritional status of patients. All body composition data were obtained in the instrument by inner software and recorded on the result sheet immediately after measurement. The result provides a plot of reactive and resistive components of the measured impedance at each frequency, as well as body weight (W), fat-free mass (FFM), total body water (TBW), extracellular water/total body water (ECW/TBW), fat mass, body cell mass (BCM), skeletal muscle mass (SMM), phase angle (PhA) and body mass index (BMI). Biological parameters include total protein (TP), albumin (ALB), pre-albumin (PA), lymphocyte count, hemoglobin (Hb), and C-reactive protein (CRP).

### Immune Cells and Inflammatory Cytokines

About 5 mL venous blood of each patient was collected in a heparin anticoagulation vacuum blood collection tube. The numbers of white blood cell (WBC), T lymphocyte (TLC), CD4^+^ T lymphocyte, CD8^+^ T lymphocyte, and neutrophil in the blood samples were measured with the MultiTEST IMK Kit (BD) by the single platform on FACSCalibur™ flow cytometry (BD). CD4/CD8 ratio and neutrophil/lymphocyte ratio (NLR) were calculated accordingly.

5 mL of fasting venous blood was collected before and after three months of nutritional intervention using blood collection vessels containing procoagulants, centrifuged at 3000 r/min for 10 min, and the upper serum was extracted and stored at -80°C. The concentrations of tumor necrosis factor-β (TNF-β), TNF-α, interleukin 1α (IL-1α), IL-6, IFN-γ inducible protein 10 (IP-10), CD14, monocyte chemotactic protein 1 (MCP-1), D-dimer, and C-reactive protein (CRP) were detected by the MILLIPLEX analysis system. IL-1β was measured by using LEGEND MAX™ Human IL-1β ELISA Kit.

### Determination of Intestinal Barrier Damage Markers

The levels of diamine oxidase (DAO), D-lactic acid (D-lactate), and lipopolysaccharide (LPS) in blood were quantitatively detected by the JY-DLT intestinal barrier function biochemical index analysis system and diamine oxidase/lactate/bacterial endotoxin detection kit (enzymatic method) (Beijing Zhongjin Golden Field).

### Statistical Analysis

All parameters post-intervention were compared with the corresponding baseline levels. Data were analyzed using a statistical software package for Windows (SPSS version 21, SPSS Inc, Chicago, Ill) and Graphpad Prism 8.1, and all statistics were analyzed by a two-sided test with a test level of alpha = 0.05. Measurement data were tested for homogeneity of variance by *Levene* method, normal distribution by *Shapiro-Wilk (S-W)* test, normal data were expressed by Mean ± standard deviation (M ± SD), and non-normal distribution measurement data were expressed by M (P25, P75). Statistical tests were performed using a paired *t*-test or *Wilcoxon* test.

## Results

### Patient Characteristics

**Figure 1:**
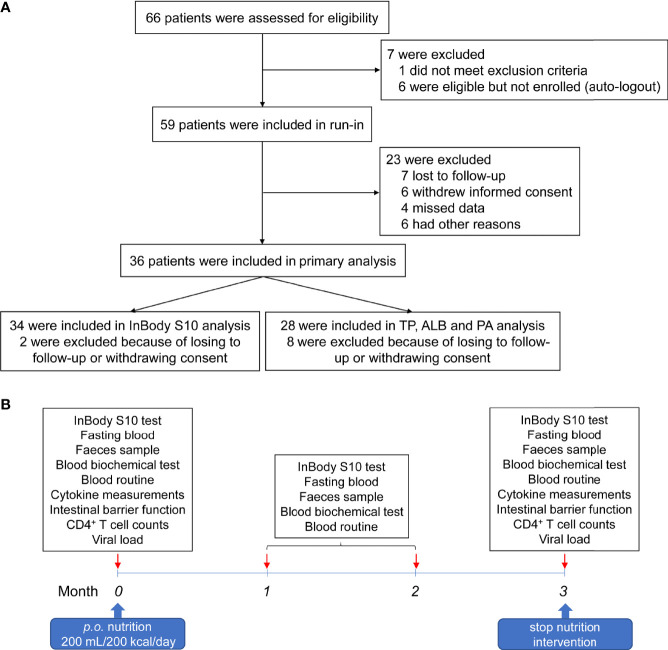
Workflow of the patient recruitment and nutrition intervention procedure. **(A)** Flow diagram of patient recruitment in the trial. **(B)** The procedures of nutritional intervention, sampling, and analyses.

From July 04, 2019 to November 07, 2019, 66 HIV-infected INRs were recruited in the Second People’s Hospital of Dali City. Seven cases were not enrolled, of whom one met exclusion criteria and six were eligible, but not enrolled as auto-logout. Fifty-nine patients underwent nutritional intervention. Three months later, seven lost to follow-up, six withdrew from the trial, four lost data, and six did not receive the trial results for other reasons (**Figure 1A**). Therefore, 36 subjects were subjected to the nutrition intervention trial (**Figure 1B**). The characteristics of these HIV-infected INRs were shown in **Table 1**.

**Table 1:** Demographic characteristics of participants.

	Participants (N = 36)
Gender, N (%)	
Male	18 (50**%**)
Female	18 (50**%**)
Age	48 ± 8
Ethnicity, N (%)	
Han	13 (36.1**%**)
Other minorities	23 (63.9**%**)
Acquisition of HIV, N (%)	
Injection drug use	11 (30.6**%**)
Sexual route	23 (63.9**%**)
Unknown	2 (5.5**%**)
Time on ART (years)	8 ± 3
CD4^+^ T cell counts (cells/μL)	208 ± 69
CD8^+^ T cell counts (cells/μL)	506 ± 295
CD4/CD8 ratio	0.53 ± 0.06
HBV and/or HCV infection, N (%)	
HBV infection	4 (11.1**%**)
HCV infection	12 (33.3**%**)
HBV and HCV co-infection	1 (2.7**%**)

Data are presented as Mean ± SD unless otherwise indicated.

HIV, human immunodeficiency virus; ART, antiretroviral therapy; HBV, hepatitis B virus; HCV, hepatitis C virus.

Of 36 enrolled patients, 50% were male and 50% were female, with an average age of 48 years. Among them, 13 (36.1%) were Han and 23 (63.9%) were of other nationalities. Among all HIV-infected patients, 30.6% were infected by intravenous drug use, 63.9% were transmitted through sexual behavior, and 5.5% were not clear about the route of infection. The average ART time of these AIDS patients was 8.07 ± 2.78 years. After over two years of ART, the average number of CD4^+^ T cells reached 208 ± 69 cells/μL and CD8^+^ T cell counts were 506 ± 295 cells/μL. In addition, 11.1% of these patients were complicated with hepatitis B virus (HBV) co-infection, 33.3% with HCV co-infection, and 2.7% of patients were co-infected with HBV and HCV.

### CD4 Cell Count and Serum Level of Inflammatory Cytokines

**Figure 2:**
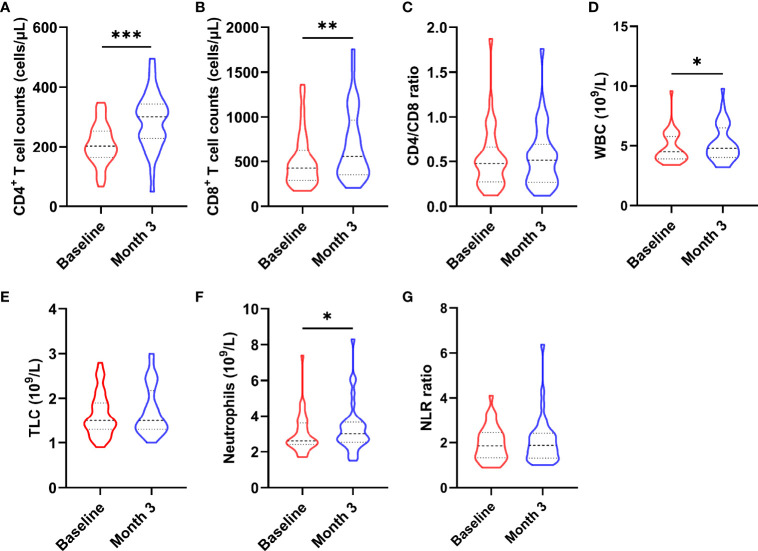
Changes in immune cells after the nutritional intervention. **(A)** CD4^+^ T cell count, **(B)** CD8^+^ T cell count, **(D)** WBC, and **(F)** neutrophils were significantly increased, while **(C)** CD4/CD8 ratio, **(E)** TCL, and **(G)** NLR did not change significantly. Statistically significant differences are indicated as *P < 0.05, **P < 0.01, and ***P < 0.001.

To determine whether nutritional intervention could help HIV-infected patients with poor immune reconstitution to restore immune function, we compared CD4^+^ T cell count, CD8^+^ T cell count, CD4/CD8 ratio, total lymphocyte (TLC), white blood cell (WBC), neutrophil and neutrophil/lymphocyte ratio (NLR) before and after intervention (as shown in **Figure 2**). The results showed that CD4^+^ T cell count (z = -4.093, *P* < 0.0001, **Figure 2A**), CD8^+^ T cell count (z = -3.780, *P* = 0.001, **Figure 2B**), WBC (t = -2.538, *P* = 0.016, **Figure 2D**), and neutrophil (t = -2.322, *P* = 0.026, **Figure 2F**) were significantly increased in AIDS patients after nutritional intervention. However, no significant changes were observed in CD4/CD8 ratio (z = -0.566, *P* = 0.572, **Figure 2C**), TLC (t = -1.406, *P* = 0.169, **Figure 2E**) and NLR (z = -0.573, *P* = 0.566, **Figure 2G**) (*P* > 0.05).

**Figure 3:**
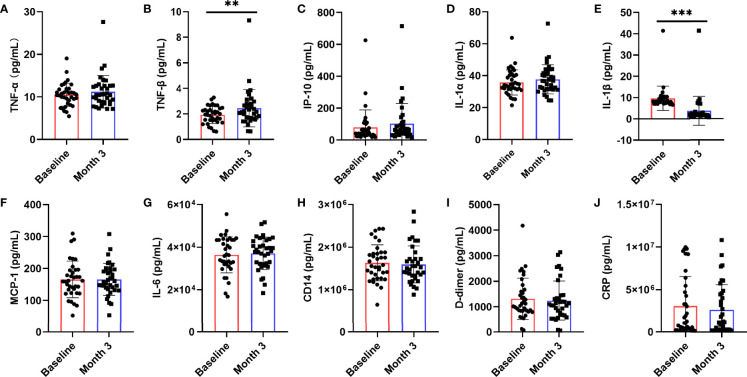
Changes in serum levels of cytokines after the nutritional intervention. After nutritional treatment, only the IL-1β **(E)** level decreased significantly, and the serum TNF-β **(B)** level was significantly higher than that before nutritional intervention. Serum levels of **(A)** TNF-α, **(C)** IP-10, **(D)** IL-1α, **(F)** MCP-1, **(G)** IL-6, **(H)** CD14, **(I)** D-dimer, and **(J)** CRP, were not significantly changed. Statistically significant differences are indicated as **P < 0.01, and ***P < 0.001.

Chronic inflammation is one of the characteristics of AIDS patients fail to restore immune functions. To further explore whether nutritional intervention could help alleviate chronic inflammation in patients with poor immune reconstruction, this study examined the changes of HIV-related cytokines and inflammatory factors (**Figure 3**), including IL-1α, IL-1β, TNF-α, TNF-β, IL-6, IP-10, MCP-1, CD14, D-dimer, and CRP. We observed that serum TNF-β (z = -2.694, *P* = 0.007, **Figure 3B**) level was significantly higher than that before nutritional intervention (*P* < 0.05), while only IL-1β (t = 11.413, *P* < 0.0001, **Figure 3E**) was significantly lower. There were no significant changes in TNF-α (z = -1.744, *P* = 0.081, **Figure 3A**), IP-10 (t = -1.674, *P* = 0.103, **Figure 3C**), IL-1α (t = -1.786, *P* = 0.083, **Figure 3D**), MCP-1 (z = -0.353, *P* = 0.724, **Figure 3H**), IL-6 (z = -1.037, *P* = 0.300, **Figure 3F**), CD14 (z = -1.097, *P* = 0.272, **Figure 3G**), D-dimer (t = 0.762, *P* = 0.451, **Figure 3I**), and CRP (t = 0.900, *P* = 0.374, **Figure 3J**) after nutritional intervention.

### Intestinal Barrier Function and Nutritional Status

**Figure 4:**
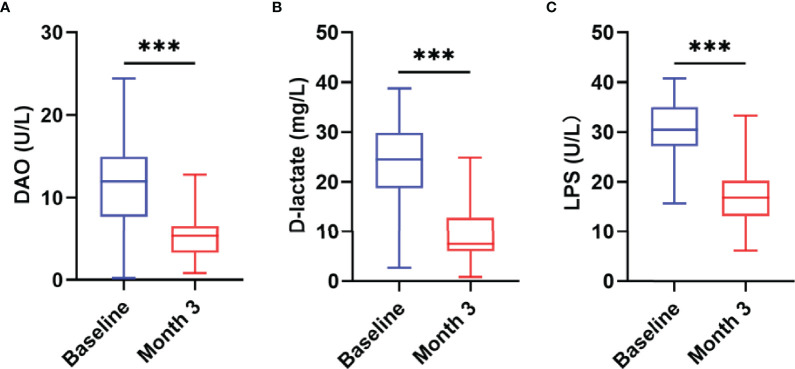
Changes in intestinal barrier function after the nutritional intervention. The serum levels of DAO **(A)**, D-lactate **(B)**, and LPS **(C)** were significantly decreased in AIDS patients with poor immune reconstitution after three months of nutritional treatment. Statistically significant differences are indicated as ***P < 0.001.

Studies have shown that there is persistent intestinal barrier damage in AIDS patients. To explore whether nutritional intervention is beneficial for the repair of the intestinal barrier, this study used the JY-DLT intestinal barrier function biochemical index analysis system to determine blood markers of intestinal barrier damage, including DAO, D-lactate, LPS. The results showed that the serum levels of DAO (*P* = 0.0019, **Figure 4A**), D-lactate (*P* < 0.0001, **Figure 4B**), and LPS (*P* < 0.0001, **Figure 4C**) were significantly decreased in AIDS patients with poor immune reconstitution after 3 months nutritional treatment compared with those before the nutritional intervention.

**Figure 5:**
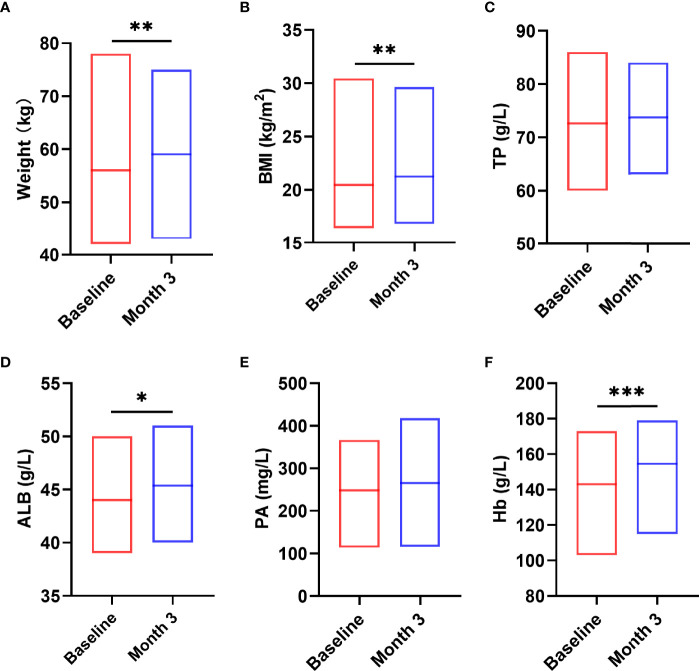
Changes in nutritional status after the nutritional intervention. After pre-digested enteral nutritional supplements were given, the nutritional indicators of patients, including W **(A)**, BMI **(B)**, ALB **(D)**, Hb **(F)**, were significantly increased. TP **(C)** and PA **(E)** were not significantly changed. Statistically significant differences are indicated *P < 0.05, **P < 0.01, and ***P < 0.001.

Next, we analyzed the indicators that can reflect the nutritional status of patients before and after nutritional intervention and related indicators of human body composition. The results showed that after 3 months nutritional treatment, the nutritional indicators of patients changed significantly, including weight (t = -3.491, *P* = 0.001, **Figure 5A**), BMI (z = -3.162, *P* = 0.002, **Figure 5B**), ALB (t = -2.269, *P* = 0.031, **Figure 5D**), hemoglobin (Hb) (t = -5.202, *P* < 0.0001, **Figure 5F**) were significantly increased compared with those before nutritional intervention. TP (t = -0.855, *P* = 0.400, **Figure 5C**) and PA (t = -1.500, *P* = 0.816, **Figure 5E**) were not significantly changed compared with those before nutritional intervention (*P* > 0.05).

**Table 2** provides a comparison of the InBody S10 data before and after intervention with enteral nutritional supplement. The results demonstrated that ECW/TBW (z = -3.576, *P* < 0.0001), BMC (z = -4.137, *P* < 0.0001), and fat content (z = -2.162, *P* = 0.031) were significantly higher than those before nutritional intervention. In contrast, the total body water/Fat free mass (TBW/FFM) (z = -3.496, *P* < 0.0001) and skeletal muscle mass index (SMI) (z = -2.732, *P* = 0.006) were significantly lower than those before nutritional intervention. Intracellular water (ICW) (t = -0.054, *P* = 0.957), extracellular water (ECW) (t = -1.518, *P* = 0.139), TBW (t = -0.608, *P* = 0.548), FFM (t = -0.806, *P* = 0.426), soft lean mass (SLM) (t = -0.451, *P* = 0.655), SMM (t = -0.608, *P* = 0.548), BCM (t = -0.060, *P* = 0.952), percentage body fat (PBF) (t = -0.855, *P* = 0.399), and PhA (t = -1.830, *P* = 0.067) were not significantly changed (*P* > 0.05).

**Table 2:** Changes of human body composition before and after nutritional intervention [Mean ± SD or M (P_25_,P_75_)].

	Baseline	Month 3	t/z	*P* value^a^
ICW (L)	21.76 ± 3.47	21.78 ± 3.65	-0.054	0.957
ECW (L)	12.69 ± 2.01	12.94 ± 2.13	-1.518	0.139
TBW (L)	34.46 ± 5.46	34.73 ± 5.76	-0.608	0.548
ECW/TBW (%)	36.85 (36.30 ~ 37.55)	37.33 (37.00 ~ 37.90)	-3.576	<0.001***
FFM (kg)	46.89 ± 7.50	47.40 ± 7.87	-0.806	0.426
SLM (kg)	44.45 ± 7.05	44.70 ± 4.77	-0.451	0.655
SMM (kg)	26.39 ± 4.53	26.40 ± 4.77	-0.033	0.974
BCM (kg)	31.17 ± 4.98	31.20 ± 5.23	-0.060	0.952
BMC (kg)	2.45 (2.05 ~ 2.74)	2.68 (2.33 ~ 3.03)	-4.137	<0.001***
Fat (kg)	9.69 (5.20 ~ 13.35)	10.09 (6.55 ~ 13.00)	-2.162	0.031*
PBF (%)	16.65 ± 8.72	17.55 ± 9.11	-0.855	0.399
PhA (°)	6.75 (5.95 ~ 6.95)	6.38 (5.80 ~ 6.85)	-1.830	0.067
TBW/FFM (%)	73.51 (73.30 ~ 73.60)	73.28 (73.07 ~ 73.52)	-3.496	<0.001***
SMI	8.12 (7.10 ~ 8.65)	7.74 (7.10 ~ 8.42)	-2.732	0.006**

a *P< 0.05; **P< 0.01; ***P< 0.001.

ICW, intracellular water; ECW, extracellular water; TBW, total body water; SLM, soft lean mass; FFM, fat-free mass; SMM, skeletal muscle mass; PBF, percent body fat; BCM, body cell mass; BMC, bone mineral content; PhA, phase angle; SMI, skeletal muscle (mass) index.

### Correlation Between Intestinal Barrier Function and CD4 Cell Count or Inflammatory Cytokines

**Figure 6:**
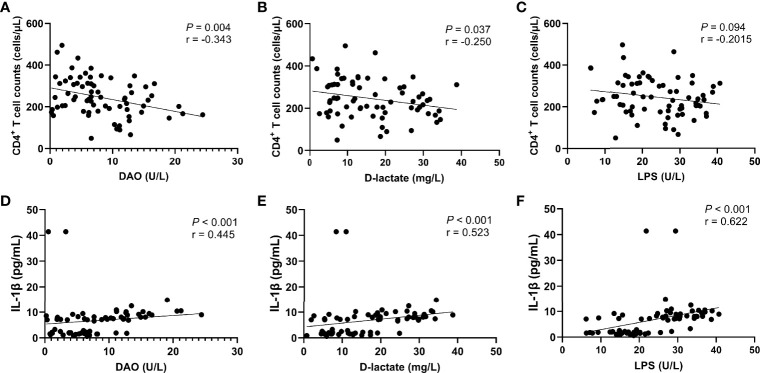
Correlation between intestinal barrier function markers and CD4^+^ T cell count or IL-1β level. CD4^+^ T cell counts were significantly negatively correlated with the serum levels of DAO **(A)** and D-lactate **(B)**, but not significantly negatively correlated with the serum level of LPS **(C)**. The IL-1β level was significantly positively correlated with the serum levels of DAO **(D)**, D-lactate **(E)**, and LPS **(F)**.

The further correlation analysis found that the levels of intestinal barrier damage markers DAO (r = -0.448, *P* = 0.002, **Figure 6A**) and D-lactate (r = -0.457, *P* = 0.001, **Figure 6B**) in peripheral blood were significantly negatively correlated with the recovery of CD4^+^ T cells, which was not observed in LPS (**Figure 6C**). In addition, we found that the levels of DAO (r = 0.420, *P* = 0.004, **Figure 6D**), D-lactate (r = 0.604, *P* < 0.0001, **Figure 6E**) and LPS (r = 0.759, *P* < 0.0001, **Figure 6F**) were significantly positively correlated with the IL-1β level.

## Discussion

In this nutritional intervention trial, we showed that the WBC, neutrophils, and CD4^+^ T cells increased significantly after three months of nutritional intervention. Overall, the nutritional status of the patients was significantly improved as well. In terms of cytokines, serum levels of IL-1β in patients after nutritional intervention decreased significantly. However, we observed that the TNF-β level increased significantly after nutritional intervention. In addition, serum levels of DAO, D-lactate, and LPS were significantly decreased in patients undergoing nutritional intervention.

CD4^+^ T cell count and CD4/CD8 ratio serve as a valid predictor of immune reconstitution prognosis after ART (20, 21). Studies have shown that there is a significant correlation between the recovery of CD4^+^ T cells and malnutrition in AIDS patients with poor immune reconstitution after ART (22–25). Malnutrition and HIV play a synergistic role in reducing the number of CD4^+^ T and CD8^+^ T cells (26), delaying skin sensitivity, reducing bactericidal performance (27), and impairing serum immunological response.

In our study, we found that after the nutritional intervention, WBC, neutrophils, and CD4^+^ T cell counts were significantly increased, while CD4/CD8 ratio did not change significantly with showing an increasing trend, suggesting that pre-digested enteral nutritional supplement could help the recovery of CD4^+^ T cells in patients with poor immune reconstitution. Ezeamama et al. has shown that HIV/AIDS patients receiving ART could benefit from nutritional support, especially for the recovery of CD4^+^ T cells (28). Pereira Da Silva et al. found that nutritional intervention could improve the nutritional status of HIV/AIDS patients by increasing the number of lymphocytes (29). A retrospective study also found a 4.7% increase in CD4^+^ T cell counts in patients after the use of nutritional supplements (30). All the above data indicated that adequate nutrition contributes to the reconstitution of immune function in HIV/AIDS patients.

This study also found that CD8^+^ T cells in the peripheral blood of patients with poor immune reconstitution remained elevated after nutritional treatment, which may be the result of persistent immune activation and inflammatory response caused by microbial translocation (31). In addition, CD8^+^ T cell counts increased, possibly because CD8^+^ T cells responded to both viral and intestinal CD4^+^ T cell depletion, and low CD4/CD8 ratio (< 1), which was not altered by nutritional intervention, promoted an increase in CD8^+^ T cell counts (32).

ART could significantly reduce systemic inflammation and immune activation, but it cannot reach the level synchronized with the HIV-uninfected population (33). Especially in patients with poor immune function reconstruction, immune activation and chronic inflammation are persistent, which may be due to malnutrition (34). Malnutrition promotes the phenotypic transformation of immune cells to pro-inflammatory phenotypes (35, 36), secreting pro-inflammatory cytokines such as IL-1β, IL-6, IL-8, TNF-α, and IL-2, while anti-inflammatory cytokines such as IL-1 receptor antagonists, IL-4, IL-10, and IL-13 decrease (37). Malnutrition is more prevalent in AIDS patients with poor immune reconstitution, which may mean that the chronic inflammatory state of patients with poor immune reconstitution is more affected by malnutrition.

High expression of IL-1β is related to HIV replication, at the same time, HIV infection could induce CD4^+^ T cell death in lymphoid tissues through caspase-1-mediated apoptosis and releasing cytokines such as IL-1β and IL-18 (38, 39). Therefore, the sustained high expression of IL-1β may be one of the reasons for poor immune reconstruction in AIDS patients. Through nutritional therapy, the level of IL-1β was significantly reduced, suggesting that nutritional intervention could reduce the inflammatory response by inhibiting HIV replication, and is beneficial in inhibiting HIV-induced CD4^+^ T cell death.

An earlier study has identified the involvement of TNF and IL-1 in HIV viral replication and pathogenesis (40). A prospective cohort study showed that TNF-α production increased in HIV patients receiving ART and increased as the disease progressed (41). There were no significant changes in TNF-α and IL-1α in enteral nutritional treatment except that IL-1β was significantly decreased, which may be due to persistent viral replication in the viral reservoirs of patients with poor immune reconstitution. In addition, TNF-β could activate macrophages and is responsible for cell-mediated immune and phagocyte-dependent protective responses (42). TNF-β is also recognized as a mediator of antigen-induced cell death (AICD) in HIV infection and stimulates HIV replication in T cells and monocyte-derived macrophages (MDM) (43). In our experiments, it is not clear which of these two conditions is responsible for the sustained rise of TNF-β, and further experiments are needed to explore.

The pathogenesis of malnutrition in HIV/AIDS patients is multifactorial, which is mainly related to changes in caloric intake, intestinal injury, nutrient absorption, and increased metabolic demand caused by active infection (8, 44). Among the many factors that cause malnutrition, impairment of intestinal function is associated with immune activation and persistent inflammatory state in AIDS/HIV patients. Damage to the mucosal barrier, microbial translocation, and immune activation during HIV infection may persist even in patients who have successfully received antiretroviral therapy (45). Intestinal barrier dysfunction could lead to increased inflammation, which further impairs the intestinal barrier by disrupting tight junction proteins. Disruption of the intestinal barrier is associated with inflammation of adipose tissue in different disease states, such as obesity, diabetes, HIV infection, and inflammatory bowel disease (46). Therefore, repairing the intestinal barrier after the nutritional intervention may be more conducive to the absorption of nutrients and the recovery of immune function and alleviate chronic inflammation.

After three months of nutritional intervention, we found that the levels of DAO, D-lactate, and LPS were significantly decreased in HIV/AIDS patients with immune non-response, suggesting that the intestinal barrier damage was repaired. The nutrients we used were supplemented with both fish oil and glutamine. Previous studies have also shown that the use of fish oil and glutamine supplements in HIV-infected individuals not only improves muscle protein consumption atrophy but also reduces inflammation and intestinal permeability (47, 48).

The levels of circulating LPS, DAO, and other intestinal barrier damage markers were significantly increased in HIV-infected patients compared with the uninfected control group (49–51). These changes lead to an increase in pro-inflammatory cytokines such as TNF-α, IL-1β, and IL-6, thereby triggering an inflammatory response (52), which is sustained by further infiltration of immune cells (e.g. neutrophils and monocytes) in response to this pro-inflammatory environment (53). Therefore, we analyzed the correlation between intestinal barrier damage and inflammatory cytokines and T cells. The results showed that CD4^+^ T cell counts were significantly negatively correlated with the levels of DAO and D-lactate, and the IL-1β level was significantly positively correlated with the serum levels of DAO, D-lactate, and LPS. This suggests that persistent intestinal barrier damage affects immune reconstitution in HIV/AIDS patients, and nutritional intervention could improve this situation.

Malnutrition is still a challenge in AIDS patients. A recent survey found that 21% of AIDS patients had mild to moderate malnutrition and 6% had severe malnutrition, and malnutrition was associated with immune non-response (25). This suggests that nutritional interventions for this population are crucial. Our data show that the weight, BMI, and Hb of patients increased significantly after enteral nutritional treatment, suggesting that nutrition intervention is beneficial for correcting the anemia of patients. Although ALB, PA, and TP did not change statistically, they all showed increasing trends, suggesting that nutritional treatment helps improve the malnutrition status of poor immune reconstitution patients. Studies on nutritional therapy for HIV/AIDS patients (13–18, 54) showed that the Hb and nutritional status of patients with nutritional therapy based on ART were improved. This is consistent with our experimental results.

In healthy people, water accounts for 50% to 70% of the body weight. It transports ingested nutrients into cells and discharges wastes out of the body (55). ICW, ECW, TBW, and ECW/TBW reflect the distribution of water in body cells. The ECW/TBW of healthy people can be maintained at a constant value (EBW/TBW = 0.38) (55, 56). After three months of enteral nutritional treatment, the distribution of intracellular and extracellular water changed, ECW/TBW increased significantly and reached normal value, suggesting that enteral nutritional treatment could help to restore the balance of intracellular and extracellular water to a certain extent, which promotes the transport of nutrients and the discharge of metabolic wastes and provides favorable conditions for the survival of cells in the body. Normally, the TBW/FFM value is 73%, representing 73% of the bodyweight without fat is body water. In our study, TBW/FFM decreased significantly after the nutritional intervention and was closer to the normal value, suggesting that the water content of defatted body weight tended to be more balanced after the nutritional intervention.

Studies have found that in addition to malnutrition due to HIV infection itself, AIDS patients also have ART-related lipodystrophy due to side effects of antiretroviral drugs (8, 57). Our study found that patients with poor immune reconstitution had lower fat and PBF, and the fat content increased significantly after nutritional intervention, while the change of PBF was not obvious. PBF and fat were positively correlated with the incidence of metabolic diseases such as diabetes, hypertension, fatty liver, cardiovascular and other diseases (58). Lower fat and PBF will lead to reduced skin insulation function, hormone disorder, and easy to lead to low immunity (59). The increase of fat content after nutritional intervention not only helps to improve the nutritional status of patients, but also could improve the immunity of patients.

However, our study found that after three months of nutritional intervention, the muscle-related indicators FFM, SLM, SMM, and BCM did not increase significantly, while SMI decreased. A previous study also showed that patients’ nutritional status improved and their fat increased after nutritional intervention, but their muscle mass did not increase, which is consistent with our results (29), suggesting that nutritional intervention alone may have a limited effect on muscle growth.

Taken together, in our study, we explored the clinical effects of a pre-digested enteral nutritional supplement in HIV-infected immunological non-responders. We found that after three months of pre-digested enteral nutritional supplementation, the nutritional status of patients was improved, and importantly, CD4^+^ T cell counts were significantly increased. At the same time, it also has a certain inhibitory effect on chronic inflammation. Further analysis showed that nutritional therapy could repair the damaged intestinal barrier, and the repair of the intestinal barrier was related to the recovery of CD4^+^ T cells and the lower level of IL-1β. The pre-digested enteral nutritional supplement is beneficial to HIV-infected INRs.

## Data Availability Statement

The dataset used in the article is available from the corresponding authors upon reasonable request.

## Ethics Statement

The study protocol was approved by the Human Clinical Research Ethics Committee of the First Affiliated Hospital of Kunming Medical University (No. 2018-L-43). Informed consent was obtained from all patients or their relatives or legally accepted representatives.

## Author Contributions

Y-QK and K-HW conceived and designed the study. S-TG, Y-XW and ZZ performed the experiments. S-TG, J-BZ, Y-XW, YX, ZZ and JG collected the data, S-TG, J-BZ, Y-XW, DL, YX and Y-QK analyzed the data. S-TG, J-BZ, DL, K-HW and Y-QK wrote and revised the manuscript. All authors have read and approved the current version of the manuscript.

## Funding

This work was partly supported by the National Natural Science Foundation of China (81660094), the Fund for Yunling Scholar (YLXL20170002), the General Joint Project of the Department of Science and Technology of Yunnan Province and Kunming Medical University (2017FE467 (-038), and 2017FE467(-130)), the Project for Innovation Team of Department of Science and Technology of Yunnan Province, China (2018HC005), the Fund of Department of Education of Yunnan Province (2019Y0352), the Fund of Health Commission of Yunnan Province (2018NS0085), the Fund of Yunnan Provincial Clinical Research Center for General Surgical Diseases (zx2019-03-03) and Yunnan Provincial Clinical Research Center for Skin Immune Diseases (2019ZF012) from Science and Technology Department of Yunnan Province, and the China Postdoctoral Science Foundation (2019M663580).

## Conflict of Interest

The authors declare that the research was conducted in the absence of any commercial or financial relationships that could be construed as a potential conflict of interest.

## Publisher’s Note

All claims expressed in this article are solely those of the authors and do not necessarily represent those of their affiliated organizations, or those of the publisher, the editors and the reviewers. Any product that may be evaluated in this article, or claim that may be made by its manufacturer, is not guaranteed or endorsed by the publisher.
