# Supplementary material for: Pre-Digested Protein Enteral Nutritional Supplementation Enhances Recovery of CD4+ T Cells and Repair of Intestinal Barrier in HIV-Infected Immunological Non-Responders
Source: Front Immunol. 2021 Dec 24;12:757935. doi: 10.3389/fimmu.2021.757935 (PMC8741150; doi:10.3389/fimmu.2021.757935)
Supplement: Supplementary file 1 [file DataSheet_1.pdf]

Supplemental materials

Pre-digested protein enteral nutritional supplementation enhances recovery of $CD4^+$ T cells and repair of intestinal barrier in HIV-infected immunological non-responders

Geng ST *et al*., Front Immunol, 2021

Table S1. Nutritional composition of the enteral nutrition formula preparation

Ingredient	Per 100 g[a]	Nutrient reference values (%)
energy	1732 kJ (414 kcal)	21%
protein	15.0 g	25%
fat	6.7 g	11%
carbohydrate	72.3 g	24%
sodium	300 mg	15%
vitamin A	500 μg RE	63%
vitamin D	1.2 μg	24%
vitamin E	7.60 mg α-TE	54%
vitamin B_1	0.90 mg	64%
vitamin B_2	1.20 mg	86%
vitamin B_6	1.50 mg	107%
vitamin B_{12}	4.50 μg	188%
vitamin C	100.0 mg	100%
nicotinamide	15.00 mg	107%
folate	425 μg DFE	106%
Pantothenic acid	2.20 mg	44%
phosphorus	80 mg	11%
potassium	620 mg	31%
calcium	80 mg	10%
zinc	7.00 mg	47%
taurine	120 mg	-

[a]The formula preparation is used with a package of 100 mg DHA and 5 g Glutamine each time.

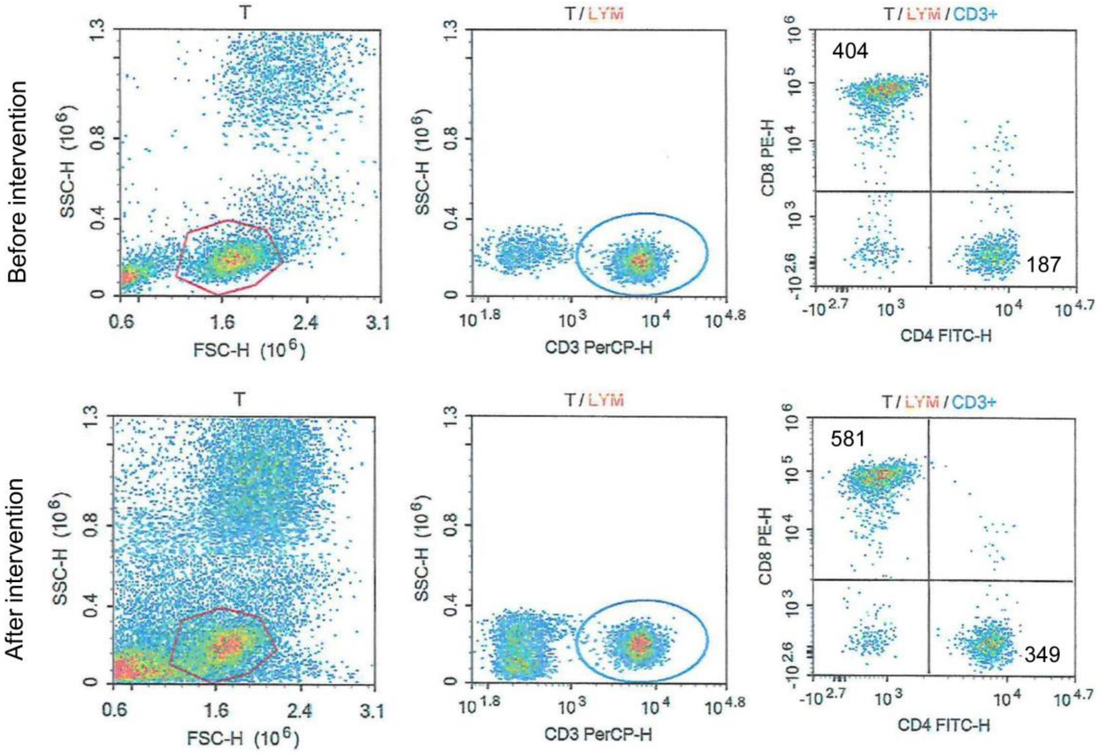

Figure S1. Flow cytometry assay of $CD3^+$, $CD4^+$ and $CD8^+$ T cell cunts. The top panel is the flow cytometric graphs before the nutritional intervention, the lower panel is the flow cytometric graphs after the nutritional intervention. The numbers in the left top grids and right low grids indicate the $CD8^+$ and $CD4^+$ T cell counts in the PBMCs of the patients, respectively.
